# New pediatric vision screener, part II: electronics, software, signal processing and validation

**DOI:** 10.1186/s12938-016-0128-7

**Published:** 2016-02-04

**Authors:** Boris I. Gramatikov, Kristina Irsch, Yi-Kai Wu, David L. Guyton

**Affiliations:** Laboratory of Ophthalmic Instrument Development, The Krieger Children’s Eye Center at the Wilmer Eye Institute, The Johns Hopkins University School of Medicine, 233, 600 N. Wolfe Street, Baltimore, MD 21287-9028 USA

**Keywords:** Vision screener, Amblyopia, Strabismus, Fixation detection, Focus detection, Birefringence, Retina

## Abstract

**Background:**

We have developed an improved pediatric vision screener (PVS) that can reliably detect central fixation, eye alignment and focus. The instrument identifies risk factors for amblyopia, namely eye misalignment and defocus.

**Methods:**

The device uses the birefringence of the human fovea (the most sensitive part of the retina). The optics have been reported in more detail previously. The present article focuses on the electronics and the analysis algorithms used. The objective of this study was to optimize the analog design, data acquisition, noise suppression techniques, the classification algorithms and the decision making thresholds, as well as to validate the performance of the research instrument on an initial group of young test subjects—18 patients with known vision abnormalities (eight male and 10 female), ages 4–25 (only one above 18) and 19 controls with proven lack of vision issues. Four statistical methods were used to derive decision making thresholds that would best separate patients with abnormalities from controls. Sensitivity and specificity were calculated for each method, and the most suitable one was selected.

**Results:**

Both the central fixation and the focus detection criteria worked robustly and allowed reliable separation between normal test subjects and symptomatic subjects. The sensitivity of the instrument was 100 % for both central fixation and focus detection. The specificity was 100 % for central fixation and 89.5 % for focus detection. The overall sensitivity was 100 % and the overall specificity was 94.7 %.

**Conclusions:**

Despite the relatively small initial sample size, we believe that the PVS instrument design, the analysis methods employed, and the device as a whole, will prove valuable for mass screening of children.

## Background

There is an increasing demand for reliable technology that remotely detects eye fixation. Of particular interest are pediatric screening devices that can detect risk factors for amblyopia (“lazy eye”). Amblyopia, that is poor development of vision in an otherwise normal eye, is a major public health problem, with impairment estimated to afflict up to 3.6 % of children—and more in medically underserved populations [1]. Amblyopia is also among the top three causes of visual impairment in adults. It can be successfully treated, but only in early childhood, especially during infancy. Delayed treatment risks lifelong visual impairment. There is thus a need for a well-validated screening device that detects the primary risk factors for amblyopia in infants and preverbal children, namely strabismus (a misaligned eye) and blurred vision (defocus). The device must require no individual calibration at these ages, and must be able to capture short-lasting intervals of central fixation [2].

There is a need for a commercially available and widely accepted automated screening instrument that can reliably detect strabismus and defocus [3]. Recent years have seen the introduction of a number of new devices intended for vision screening: PlusOptix S12C and predecessors (PlusOptix, Atlanta, GA), Spot Vision Screener (Welch Allyn, Skaneateles Falls, NY), 2WIN (Adaptica, Padova, Italy), iScreen Vision 3000 from iScreen Vision Inc. (Cordova, TN), and others. These devices are basically photoscreeners that use cameras to detect asymmetry of light reflections from the eyes to estimate misalignment, and also estimate the refractive error of each eye by photo-retinoscopy, a variation of the Foucault knife-edge test on light retro-reflected from the fundus of the eye. However, these devices have problems of their own that constrain their effectiveness, especially when judged against the high level of performance (e.g. sensitivity and specificity) required to justify screening in the current environment of limited health care resources. A particular problem has been low accuracy in detecting eye alignment/misalignment by using only reflections from the external surfaces of the eye, without directly sensing the projections of the actual retinal fixation points into space.

**Fig. 1:**
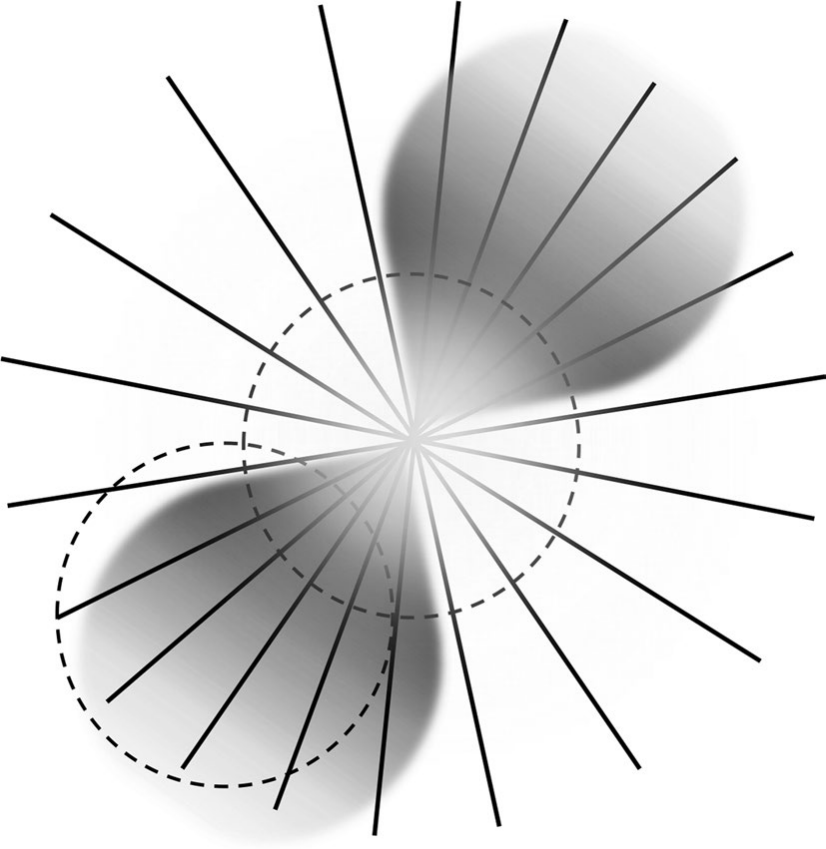
The human fovea (the small area of the retina responsible for sharp central vision) surrounded by uniquely arranged nerve fibers (Henle fibers). These fibers transmit the information to the optic disc and to the brain. The Henle fibers are birefringent, causing polarization change in the reflected light. The polarization change depends on the angle between the orientation of the fiber and the plane of polarization of the incoming light. Shown is the 3° scanning circle. When the center of the fovea coincides with the center of the scanning circle, central fixation is attained and detected. In the simplest case, during central fixation, the frequency of the returning signal would be twice the scanning frequency. Note that it is the fovea that is moving, and not the scanning circle. Retinal birefringence scanning (RBS) is a technique that uses the changes in the polarization of light returning from the eye to detect the projection into space of the array of Henle fibers surrounding the fovea

Our laboratory has been developing novel technologies for detecting accurate eye alignment directly, by exploiting the birefringence (property that changes the polarization state of light) of the uniquely arranged nerve fibers (Henle fibers) surrounding the fovea (the small area of the retina responsible for sharp central vision)—Fig. 1. Retinal birefringence scanning (RBS) is a technique that uses the changes in the polarization of light returning from the eye to detect the projection into space of the array of Henle fibers surrounding the fovea [4, 5]. In RBS, polarized near-infrared light is directed onto the retina in a circular scan, with a fixation point in the center, and the polarization-related changes in light retro-reflected from the ocular fundus are analyzed by means of differential polarization detection. Due to the radially symmetric arrangement of the birefringent Henle fibers, a characteristic frequency appears in the obtained periodic signal when the scan is centered on the fovea, indicating central fixation. For example, in the simplest possible type of analysis, when scanning around the center of the fovea, one would obtain a signal of frequency twice the scanning frequency, whereas a para-central scan would return a signal of just the scanning frequency (Fig. 1). By analyzing frequencies in the RBS signal from both eyes simultaneously, the goodness of eye alignment can be measured, and thus strabismus can be detected. RBS technology is the only known technology that can detect central fixation remotely using true anatomical information (position of the fovea). An early version of the “Pediatric Vision Screener” (PVS) was designed in our lab and tested at Children’s Hospital, Boston [6–9]. This is being developed into a commercial prototype that detects eye alignment but not defocus (REBIScan, Boston, MA). In a recent study, this prototype was compared with the SureSight Autorefractor. Its sensitivity to detect strabismus and amblyopia (0.97; 95 % CI 0.94–1.00) was significantly higher than that of the SureSight Autorefractor (0.74; 95 % CI 0.66–0.83). The specificity of the REBIScan screener for strabismus and amblyopia (0.87; 95 % CI 0.80–0.95) was significantly higher than that of the SureSight Autorefractor (0.62; 95 % CI 0.50–0.73) [10]. Meanwhile, development of the RBS technology continued in our lab, resulting in a series of central fixation detecting devices with no moving parts [11, 12], devices for *continuous* monitoring of fixation [13], a device for biometric purposes [14], and ultimately an improved PVS that combines “wave-plate-enhanced RBS” [15], or “polarization-modulated RBS” [16, 17], for detecting strabismus, with added technology for assessing proper focus of both eyes simultaneously. Polarization-modulated RBS is an optimized upgrade of RBS, based upon our theoretical and experimental research and computer modeling, using a spinning half wave plate (HWP) and a fixed wave plate (WP) to yield high and uniform signals across the entire population. In addition, using phase-shift-subtraction (described below), the new PVS eliminated the need for initial background measurement [15–17].

The present paper describes the electronics and the analysis algorithms of our new PVS. The objective of this study was to optimize the decision making and validate the performance of this research instrument that also serves as a prototype for a screening device for amblyopia risk factors in young children.

## Methods

### Central fixation (CF) detection

The retina is scanned with a low-power focused beam of linearly polarized light. Upon reflection from different points surrounding the fovea, polarization is changed differently. After certain optimization using a polarization-manipulating WP (optical retarder), the S_1_ Stokes component [18, 19] can satisfactorily describe the polarization change and yield an RBS signal. In the Stokes vector representation of the polarization state of light, **S** = [S_0_, S_1_, S_2_, S_3_], S_1_ represents the intensity difference between the horizontal (*p*) and the vertical (*s*) linearly polarized components [18, 19]. In our earlier devices we used a polarizing beam splitter (PBS) to measure the *s*- and *p*-components separately, before building the difference electronically. This, however, required two photodetectors for each eye, and extremely precise balancing, before building the difference in analog differential stages. An alternative would have been to complete the whole analog chain separately for the *s*- and *p*-components (photodetectors-filters-amplifiers), digitize separately, and then build the difference in software, thus doubling the size of the hardware. In the present design, we use a double-pass spinning HWP, which rotates the axis of linear polarization by twice the angle between its fast axis and the input’s plane of polarization. Due to this property of polarization rotation, the spinning HWP enables measurement of the two orthogonal polarization states with the same photodetector at different points in time, thereby avoiding errors associated with gain mismatch. Using a computer model involving all polarization-changing components of the system, including the Henle fibers and the cornea, we found that by spinning the HWP 9/16ths as fast as the circular scan, strong signals are generated that are odd multiples of half of the scanning frequency [17]. With central fixation, two frequency components predominate the RBS signal: 2.5 or 6.5 times the scanning frequency *f*_s_, depending on the corneal birefringence. With paracentral fixation, these frequencies practically disappear, being replaced by 3.5 and 5.5 *f*_s_. Therefore, the relative strengths of these four frequency components in the RBS signal distinguishes between central and paracentral fixation.

**Fig. 2:**
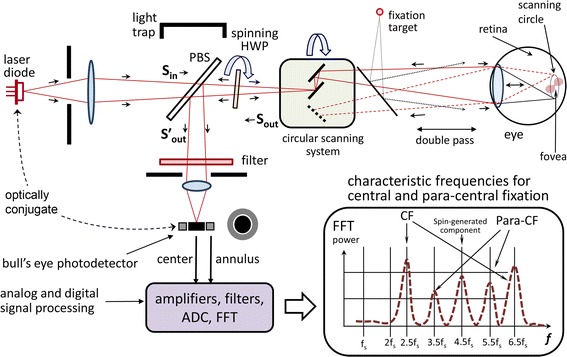
A simplified diagram of the pediatric vision screener (shown is one eye only). Linearly polarized light emitted continuously by a 785-nm laser diode is transmitted by a plate polarizing beamsplitter (PBS) toward a half-wave-plate (HWP) that is spun by a motor using a pulley ratio to achieve a rotation 9/16ths as fast as the scan. After passage through the rotating HWP, the beam of continuously rotating linearly polarized light enters the scanning unit that consists of two gold-plated plane mirrors. The retina is scanned by the spot of laser light in a circle subtending a visual angle of 3° in diameter. A small percentage of light reflected from each ocular fundus is re-imaged back, following the same light path it originally came from, via the principle of conjugacy. The unchanged part of the returning light, in other words the part with the same polarization as the original light, is transmitted through the PBS, back toward the light source, thus never making it to the detection unit. The changed part of the returning light, on the other hand, is reflected by the PBS toward the photodetector assembly, consisting of a bull’s-eye photodetector (BEPDs). A band pass filter assures that only light in the desired wavelength range reaches the detectors. The* graph* in the right-hand bottom corner shows the generated frequencies for central and paracentral fixation

The instrument design has been explained in detail elsewhere [16, 17]. A simplified diagram of the PVS for one eye only is shown in Fig. 2. Linearly polarized light emitted continuously by a 785-nm laser diode is transmitted by a plate PBS toward a HWP that is spun by a motor using a pulley ratio to achieve a rotation 9/16ths as fast as the scan. After passage through the rotating HWP, the beam of continuously rotating linearly polarized light enters the scanning unit that consists of two gold-plated plane mirrors. The scanning unit is driven by the same motor, thus turning the stationary beam of light into a circular scan. Light from the outer scanning mirror travels toward the eyes. While each eye is fixating on a blinking red light, appearing to be in the center of the scanning circle, each retina is scanned by the spot of laser light in a circle subtending a visual angle of 3° in diameter. A small percentage of light reflected from each ocular fundus is re-imaged back, following the same light path it originally came from, via the principle of conjugacy. The unchanged part of the returning light, in other words the part with the same polarization as the original light, is transmitted through the PBS, back toward the light source, thus never making it to the detection unit. The changed part of the returning light, on the other hand, is reflected by the PBS toward the photodetector assembly, consisting of two bull’s-eye photodetectors (BEPDs), one for each eye. A band pass filter assures that only light in the desired wavelength range reaches the detectors.

The fixation target is a red 690-nm laser diode flashing on and off to attract the child’s attention. The fixation light is positioned physically conjugate to the circular scan of the 785-nm laser. However, as eyes do not accommodate (focus) well on monochromatic light, we employ an improved target system with accommodative control. A vertical black-and-white grid (not shown), printed on a transparency, serves as an accommodative target, which is illuminated by a white-light LED array. This white-light accommodative background is imaged 1:1 into an aerial image plane that is 33 cm away from the subject, a standard near testing distance for children. To account for the eye’s longitudinal chromatic aberration, the image of the RBS spot of 785-nm light is located 0.75 D farther away than the 33 cm distance of the accommodative grid target, at 44.4 cm. Thus, with an eye fixating on the blinking red light, with focus controlled by the black-and-white background at 33 cm, the near-infrared light from the scanning 785-nm laser diode will be in proper focus on the retina.

### Focus detection (FD)

In the return path, the de-scanned, double-pass image of the scanning point source is projected onto the center of the BEPD. Because the scanning laser and BEPD are optically conjugate to each other with respect to the eye, and the fixation target is in the same plane as the scanning circle, all the light returning from the eye is expected to be concentrated in the center of the BEPD in the case of good focus. With poor focus, some of the returning light will spill over onto the annulus. It is known that the eye’s refraction changes little within 5° of the center of the fovea, which enables the simultaneous detection of central fixation and focus detection using the same spot of light spinning in a 3° scanning circle. Our previous studies have demonstrated a strong, spin-generated, 4.5*f* frequency in our RBS signal that is practically independent of corneal birefringence and of the position of the scanning circle with respect to the center of the fovea [16]. This “spin-generated frequency” is thus suited for assessment of the state of focus. Similar to previous studies with our BEPD [20], a normalized measure of the goodness of focus for each eye is used [(C–A)]/(C + A)], where C is the 4.5*f* signal power from the center, and A is the 4.5*f* signal power from the annulus. In contrast to the cited work, however, where the modulation was achieved by electrically turning on and off the target light, here modulation of the received light is realized by means of the spinning HWP [16]. This allowed us to simplify the design by combining CF detection with focus/defocus detection.

*The polarization-sensitive part of the instrument was optimized* using a computer model consisting of a train of Mueller matrices [11, 17, 21–23], each of which represents a stationary or rotating retarder as part of the optics, the cornea, or the Henle fiber layer of the retina, all of which change the polarization state of light. The model was optimized on a database of corneal measurements from 73 human subjects, provided by Drs. Robert Knighton and XiangRun Huang from the McNight Vision Research Center, Bascom Palmer Eye Institute, University of Miami [24] and extended with additional measurements in our lab using a research-enhanced version of the GDx-VCC instrument (courtesy of Carl Zeiss Meditec) [11, 15, 17].

**Fig. 3:**
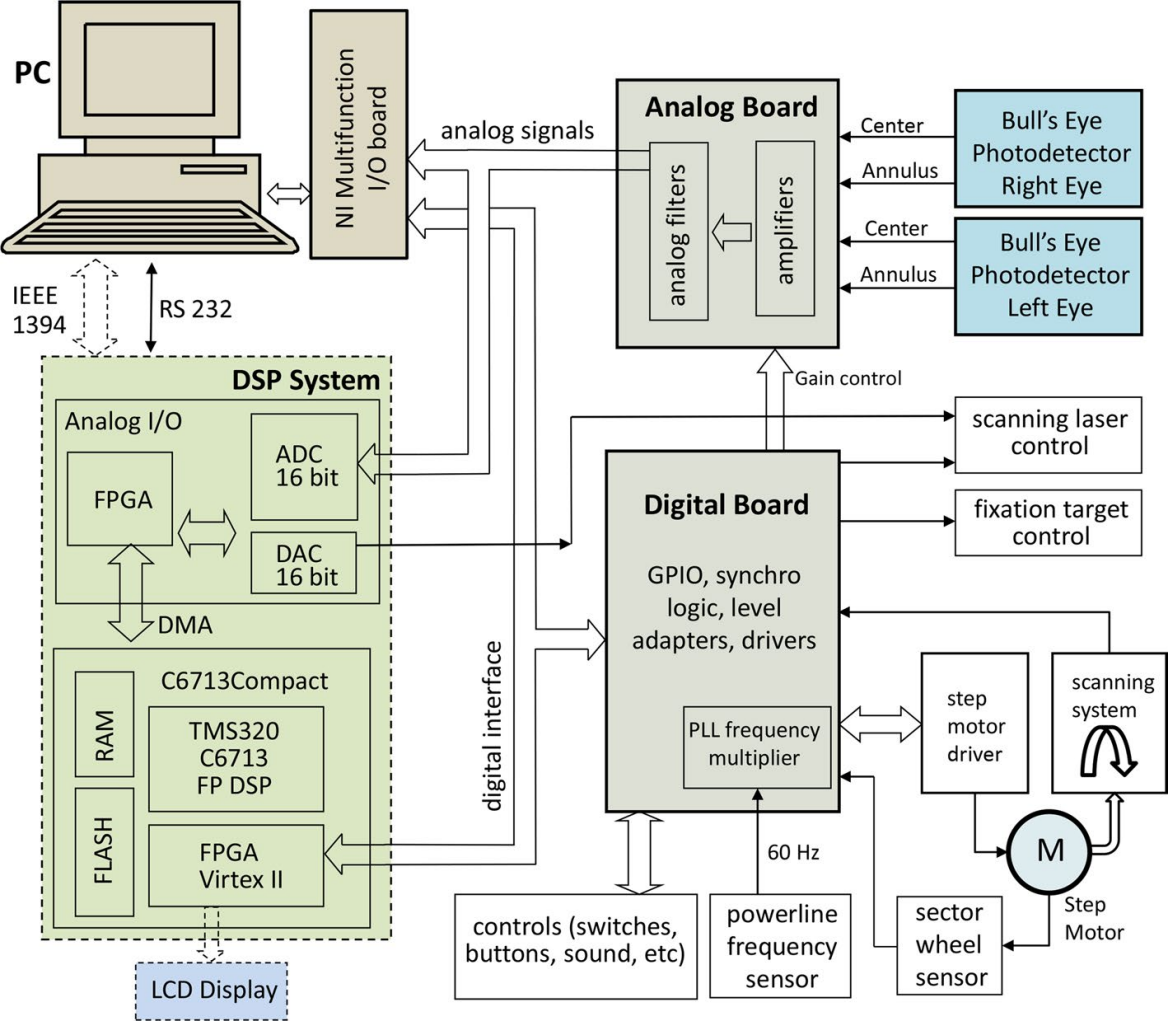
Electronics of the PVS instrument. The four signals coming from the two BEPDs (center and annulus for each eye) are amplified and filtered by a 4-channel, 4-stage programmable-gain amplifier. The amplified signals are then fed to the analog-to-digital converter (ADC). The instrument was developed with two versions of computer support: **a** a luggable (“lunchbox”) PC, and **b** a 32-bit digital signal processing (DSP) system. The first (PC-based) configuration is easier to use for development, software debugging, algorithm validation, graphical user interface, PC graphics, etc. The second (DSP-based) configuration, was designed as a CPU support for an industrial prototype, with easily reconfigurable FPGA hardware

**Fig. 4:**
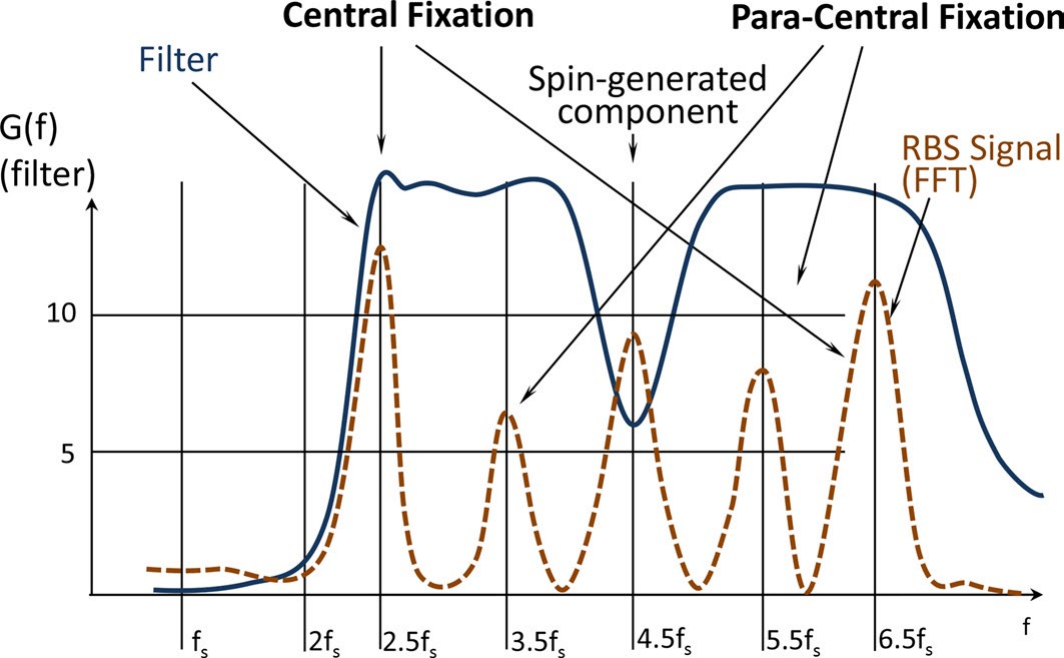
The analog filter *G(f)* of each of the four analog channels. Shown are the frequencies to be detected. Note that the 4.5 *f*_*s*_ frequency of relatively high amplitude needed less gain, to avoid saturation of the analog channels

*The electronics* of the instrument are shown in Fig. 3. The four signals coming from the two BEPDs (center and annulus for each eye) are amplified and filtered by a 4-channel, 4-stage programmable-gain amplifier with a frequency response shown in Fig. 4, along with the main frequencies to be detected. The amplified signals are then fed to the analog-to-digital converter (ADC). The instrument was developed with two versions of computer support: a) a luggable (“lunchbox”) PC with a multifunction analog/digital IO board PCI-6034E (National Instruments, 200 kS/s, 16-Bit analog I/O, 8 DIO) backed by their CVI development environment (C-language and rich GUI and hardware-support libraries), and b) a 32-bit Digital Signal Processing (DSP) system (C6713Compact, Traquair Systems), built around the TMS320C6713 chip (300 MHz floating point 1.6 G-FLOPS DSP from Texas Instruments), equipped with 16-bit analog I/O and a 0.5 M-gate field programmable gate array (FPGA), a real-time operating system (DSP/BIOS) and the TI’s chip support library (CSL). The software was developed and tested for both systems, and the PVS can be reconfigured for either of the two CPU systems. The first (PC-based) configuration is easier to use for development, software debugging, algorithm validation, graphical user interface, PC graphics, ability to function side-by-side with MATLAB, etc., and was used as the main platform for developing the PVS as a research instrument. The second (DSP-based) configuration, was designed more as a CPU support for an industrial prototype, with easily reconfigurable FPGA hardware using the ISE environment of Xilinx with VHDL language.

**Fig. 5:**
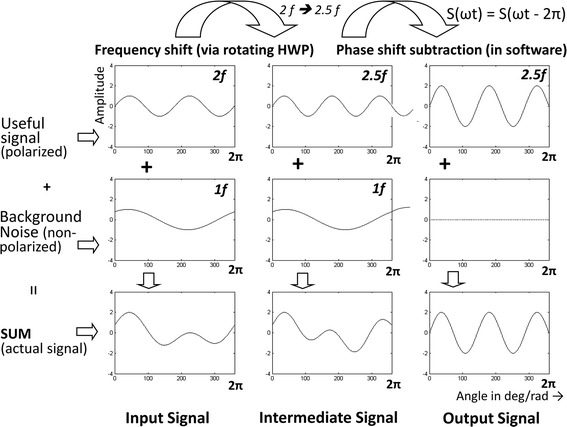
The phase-shift-subtraction method. The signal is subtracted from itself shifted by one period of the scanning system. This removes the background noise, while the useful odd multiples of half the scanning frequency (shown is only 2.5 *f*_*s*_) are doubled in amplitude

*The phase-shift-subtraction (PhSS)* method is an algorithm that we developed and implemented in order to reduce non-RBS noise in real time [16] (Fig. 5). The light that has been depolarized upon reflection by the skin and sclera (background noise), is not affected by the spinning WP, so its waveform still repeats itself over a single scanning cycle (360°) at *f*_s_. If we subtract the signal from itself shifted by one period of the scanning system T = 1/*f*_s_, the background noise will be fully removed from the signal, while each odd multiple of half the scanning frequency will be doubled in amplitude, as shown in the third column of the figure. The example is shown for frequency components of 2.5 *f*_s_, but the method works with every odd multiple of half the scanning frequency. This approach eliminates most of the background noise associated with conventional RBS in our earlier versions of the PVS (including the REBIscan device) and greatly improves the signal-to-noise ratio without the need for a preliminary background measurement and subsequent background subtraction.

**Fig. 6:**
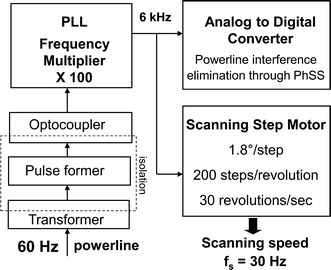
Deriving the A–D sampling rate and the step-motor pacing pulses from the power line frequency. In order to eliminate the power line interference along with the instrumental noise in the PhSS procedure, the motor was paced with a frequency which is a precise multiple of the 60 Hz power line frequency: 6000 steps/sec (100 × 60 Hz). The power line frequency was obtained by means of a transformer with 4 kV isolation. Multiplication of the power line frequency by a factor of 100 is achieved by means of a phase-locked-loop (PLL) circuit. Thus, the motor is spinning at a speed of 6000/200 = 30 rotations per second. At each new step, one sample from each channel is acquired, i.e. every new data sample corresponds to 1.8° of rotation (1 step). Each of the 200 samples during one scanning rotation corresponds to one particular angle of rotation, with the angles spaced evenly at 1.8° intervals. At the same time, there are exactly two complete power line cycles (60 Hz) in one scanning cycle (*f*_s_ = 30 rps), which means that power line noise is eliminated by the 360° PhSS technique

The PhSS method requires a very stable motor speed, as well as data sampling at precise moments in time and motor shaft angles during each scan. This was achieved with a step motor (CSK 264-BT-2, Oriental Motor USA) with 1.8°/step (200 steps per 360°). In order to eliminate the power line interference along with the instrumental noise in the PhSS procedure, we paced the motor with a frequency which is a precise multiple of the 60 Hz power line frequency: 6000 steps/sec (100 × 60 Hz). The power line frequency was obtained by means of a transformer with 4 kV isolation. Multiplication of the power line frequency by a factor of 100 is achieved by means of a phase-locked-loop (PLL) circuit. Thus, the motor is spinning at a speed of 6000/200 = 30 rotations per second (Fig. 6). At each new step, one sample from each channel is acquired, i.e. every new data sample corresponds to 1.8° of rotation (1 step). Each of the 200 samples during one scanning rotation corresponds to one particular angle of rotation, with the angles spaced evenly at 1.8° intervals. At the same time, there are *exactly* two complete power line cycles (60 Hz) in one scanning cycle (*f*_s_ = 30 rps), which means that power line noise is eliminated by the 360° PhSS technique.

*Laser safety* complied with the ANSI Z136.1 standard of the Laser Institute of America. The irradiance at the location of the pupil is 0.2 mW/cm^2^, well below the maximum permissible exposure (MPE) of 1.48 mW/cm^2^ for a wavelength of 785 nm, for ocular exposure to a stationary point source for a duration up to 30,000 s. The instrument safety was approved by the Clinical Engineering Department of Johns Hopkins University.

### Testing device operation

**Fig. 7:**
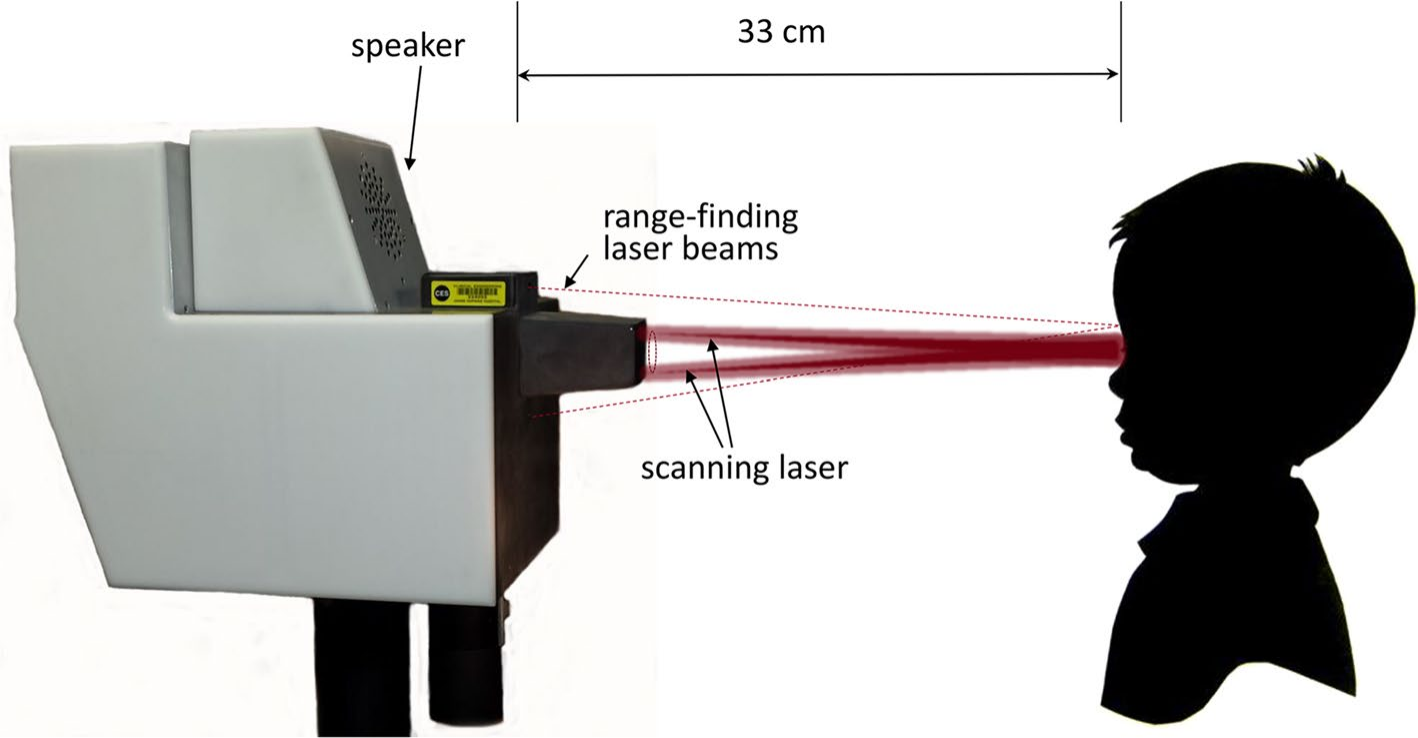
The examination procedure. Without head restraint, the child is seated on a chair or in the parent’s lap, while the operator aims the instrument from a distance of 33 cm (± 1 cm) using built-in triangulating laser pointers converging on the bridge of the nose. During the exam, the child sees the flashing fixation target within the aperture of the instrument, (accompanied by synchronous sound), and the scanning circle

Without head restraint, the child is seated on a chair or in the parent’s lap, while the operator aims the instrument from a distance of 33 cm (± 1 cm) using built-in triangulating laser pointers converging on the bridge of the nose [16]. This desired axial distance (not critical) is achieved when the spots from two aiming low power lasers overlap on the bridge of the child’s nose (Fig. 7). During the exam, the child sees the flashing fixation target within the aperture of the instrument, accompanied by synchronous sound, to encourage the child to maintain fixation on the flashing target light. Room lights are dimmed to enhance interest and to aid pupil dilation which is most expressed in the first minute after dimming [25]. The operator initiates a data acquisition session which contains a preselected number (12) of 1-second-long records, acquired and analyzed in succession. For each record, after applying the PhSS procedure, the power spectrum is computed for each eye, for each of the two photodetectors comprising each BEPD. All characteristic frequencies for all 12 records in a session are stored in RAM for final decision making, and are saved on disk at the end of the exam. For each record, each eye is declared passing for central fixation if a CF criterion (described below) is met. The necessary condition for an eye to pass for central fixation is that the CF criterion is satisfied for at least two out of 12 records (16.7 %). Proper eye alignment requires simultaneous central fixation with both the right and the left eye.

Similarly, each eye is tested for each record using a FD criterion (described below). Proper focus for an eye is declared if at least two out of 12 records satisfy the FD criterion. The instrument displays all four decisions for each record, via virtual LEDs in real time [16], and in a table in a separate *Results* window, so that the operator can follow precisely the course of the recording session. Because all measured signals are stored in raw data files, the device allows us to re-run all sessions, should the analysis rules and the decision-making logic change.

### Criterion for central fixation

As described before, RBS in the foveal region results in the generation of four distinct frequencies in the signal obtained from the returned light. These frequencies are acquired by performing FFT, and their power spectrum components are *P*_*2.5*_ and *P*_*6.5*_ for central fixation frequencies 2.5 and 6.5 *f*_*s*_ respectively, and *P*_*3.5*_ and *P*_*5.5*_ for para-central fixation frequencies 3.5 and 5.5 *f*_*s*_, respectively. Power spectrum component *P*_*4.5*_ (for 4.5 *f*_*s*_) is independent of the direction of gaze. Because all the above components depend on subject-specific factors such as pupil size, reflectivity of the retina, retinal and corneal birefringence, instrument design, etc., spectral powers *P*_*2.5*_*, P*_*6.5*_*, P*_*3.5*_ and *P*_*5.5*_ are normalized by *P*_*4.5*_, thus making them dependent mainly on the direction of gaze.

With all four spectral components available, it is still not trivial to establish a decision making logic which allows precise detection of CF or lack thereof. To find a statistically reliable solution, as part of the CF calibration procedure, we recorded signals from additional five asymptomatic normal volunteers, ages 10, 18, 24, 29 and 39, all properly consented. The subjects were asked to look first at the blinking target in the center of the scanning circle, for CF. Twelve measurements were taken for each one of them, each lasting 1 s. The raw signals, as well as the calculated FFT powers for each measurement, were saved on disk. The same type of measurement was repeated with each of the subjects looking at imaginary “targets” on the scanning circle (1.5° off center), at 12, 3, 6, and 9 o’clock. The data, consisting of powers *P*_*2.5*_*, P*_*6.5*_*, P*_*3.5*_*, P*_*5.5*_ and *P*_*4.5*_ for each of the 12 measurements of each eye of all five test subjects, were bundled into two groups: a group for central fixation (120 “eyes,” the “CF set”) and a group for para-central fixation (480 “eyes,” the “para-CF set”).

Analysis was performed in MATLAB. Two algorithms were developed for classification into one of the two classes (CF vs para-CF) using the available signal power measurements:

Method 1 employed a gradual change of the threshold for *(P*_*2.5*_ + *P*_*6.5*_*)/P*_*4.5*_ in order to optimize a single threshold for the sum of only central fixation frequencies. The threshold *θ* for decision making was increased in small steps of 0.001, starting with *θ*_min_ = 0.3 and ending with *θ*_max_ = 8.00. At each level *i*, the error for central fixation was calculated as the sum of the absolute values of the individual errors for the CF set:

1 $$E_{i}^{CF} = \sum_{j = 1}^{n\_CF} \left| {\frac{{\left( {P_{2.5}^{j,CF} + P_{6.5}^{j,CF} } \right)}}{{P_{4.5}^{j,CF} }} - \theta_{i} } \right|$$

where $\left( {P_{2.5}^{j,CF} + \, P_{6.5}^{j,CF} } \right)/ \, P_{4.5}^{j,CF}$ is the normalized sum of the two central fixation powers for each CF measurement *j*, and *θ*_i_ is the threshold at the current level *i*. Similarly, the error for para-central fixation was calculated as the sum of the absolute values of the individual errors for the para-CF set:

2 $$E_{i}^{paraCF} = \mathop \sum \limits_{j = 1}^{n\_paraCF} \left| {\frac{{\left( {P_{2.5}^{j,paraCF} + P_{6.5}^{j,paraCF} } \right)}}{{P_{4.5}^{j,paraCF} }} - \theta_{i} } \right|$$

where $\left( {P_{2.5}^{j,paraCF} + \, P_{6.5}^{j,paraCF} } \right)/ \, P_{4.5}^{j,paraCF}$ is the normalized sum of the two central fixation powers for each para-CF measurement *j*, and *θ*_i_ is the threshold at the current level *i*. The algorithm chooses the threshold *θ*_*i*_ that minimizes the total error:

3 $$E = min\left\{ {E_{i}^{CF} + E_{i}^{paraCF} } \right\}$$

Method 2 employs linear discriminant analysis, basically using a linear combination of features (in our case the normalized signal powers) to separate the two classes (CF vs para-CF). We used a linear classifier which generally provides a solution in the form

4 $$K + {\mathbf{PL}}^{\text{T}} = 0$$

where **P** is the features vector, while *K* and **L** define the coefficients *a*_*i*_ of the discriminant function. For example, a three-way linear discriminant analysis provides a plane in the form

5 $$K + \left[ {x \, y \, z} \right]{\mathbf{L}}^{\text{T}} = 0,\quad{\mathbf{P}} = \left[ {x \, y \, z} \right],\quad{\mathbf{L}} = \left[ {{\text{L}}\left( 1\right){\text{ L}}\left( 2\right){\text{ L}}\left( 3\right)} \right]$$

where *x*, *y*, and *z* stand for *(P*_*3.5*_ + *P*_*5.5*_*)/P*_*4.5*_, *P*_*2.5*_*/P*_*4.5*_, and *P*_*6.5*_*/P*_*4.5*_, respectively. Classification is based on the linear classifier

6 $${\text{z}} = a_{0} + a_{1} x + a_{2} y$$

where *a*_*0*_ = –(K/L(3)), *a*_*1*_ = –(L(1)/L(3)), and *a*_*2*_ = –(L(2)/L(3)). The measurement to be classified is considered belonging to the class of central fixation if *P*_*6.5*_*/P*_*4.5*_> z (as calculated above). The two- and four way discriminant analyses are similar and will not be discussed in detail here. The main function for discriminant analysis is classify(), part of the *Statistics and Machine Learning Toolbox*. The results obtained with Method 1 and all three cases of Method 2 are summarized in Table 1.

**Table 1:** Performance of the different methods for classifying fixation as central vs para-central

	Simple threshold (method 1)	Discriminant analysis (method 2)		
		2D	3D	4D
	$\frac{{\left( {P_{2.5} + P_{6.5} } \right)}}{{P_{4.5} }}$	$\frac{{\left( {P_{2.5} + P_{6.5} } \right)}}{{P_{4.5} }} vs \frac{{\left( {P_{3.5} + P_{5.5} } \right)}}{{P_{4.5} }}$	$\frac{{P_{6.5} }}{{P_{4.5} }} vs \frac{{P_{2.5} }}{{P_{4.5} }} vs \frac{{\left( {P_{3.5} + P_{5.5} } \right)}}{{P_{4.5} }}$	$\frac{{P_{6.5} }}{{P_{4.5} }}\, vs \frac{{P_{2.5} }}{{P_{4.5} }}\, vs \frac{{P_{3.5} }}{{P_{4.5} }}\, vs\frac{{P_{5.5} }}{{P_{4.5} }}$
	*θ* = 0.8750	*a*_*0*_ = 0.743, *a*_*1*_ = 0.318	*a*_*0*_ = 0.768, *a*_*1*_ = 0.056, *a*_*2*_ = −0.289	*a*_*0*_ = 0.779, *a*_*1*_ = 0.016, *a*_*2*_ = −0.283, *a*_*3*_ = 0.083
Sensitivity	0.9917	0.9083	0.9417	0.9417
Specificity	0.9625	0.9771	1.0000	1.0000

All methods use spectral power at odd multiples of half the scanning frequency *f*_*s*_. Method 1 uses optimized simple threshold, whereas Method 2 utilizes 2-, 3-, and 4-way linear discriminant functions, whose coefficients are shown in the table

### Determining a threshold for “passing” goodness of focus

We have reported first attempts to define the “passing” threshold for the normalized focus signal (C-A)/C + A), using performance evaluations in volunteers, mainly adults [16]. Although the threshold we chose was able to identify defocus in many children, we felt that further optimization was needed. This is usually done by determining monocular focus curves: the normalized focus signal is plotted while ophthalmic trial lenses (in 0.25 D increments), tilted to avoid reflections, are placed in front of the eye to simulate various refractive errors. This enables us to find a threshold signal level under which the subject fails the focus screen, thus identifying those subjects where the amount of optical blur is abnormal and warrants further investigation. Up to 1.00 D of spherical defocus, with an average size pupil, rarely leads to amblyopia.

Focus curves in both directions of defocus away from best focus are easy to obtain in older adults who have lost their ability to “accommodate” [16]. But when insufficient lens power is placed before children’s eyes for close focusing, the children will automatically add plus power to their crystalline lenses (“accommodate”) to compensate for the insufficient lens power, and the focus signal will fall off much more slowly in the minus direction from best focus. Cycloplegic drops can be given in children to paralyze their accommodation, but this always dilates their pupils as well, causing more blur for a given amount of refractive error, thus steeping both sides of the focus curves. We studied one 10-year old boy as a test subject, to determine a “passing” threshold for focus, using cyclopentolate 1 % for cycloplegia, with the accompanying dilation.

### *Test subjects*

The protocol followed an institutional IRB approval. Eighteen properly consented patients with known vision abnormalities (eight male and 10 female), ages 4–25 (only one above 18) and nineteen controls with proven lack of vision issues (10 male and 9 female), ages 2–37 (only four above 18), all properly consented, were recruited from the patients of the Division of Pediatric Ophthalmology at the Wilmer Eye Institute, or the patients’ siblings. All subjects underwent a vision exam by an ophthalmologist, during which eye alignment and refraction were tested and the results were documented.

## Results

### Discriminant analysis for CF criteria

**Fig. 8:**
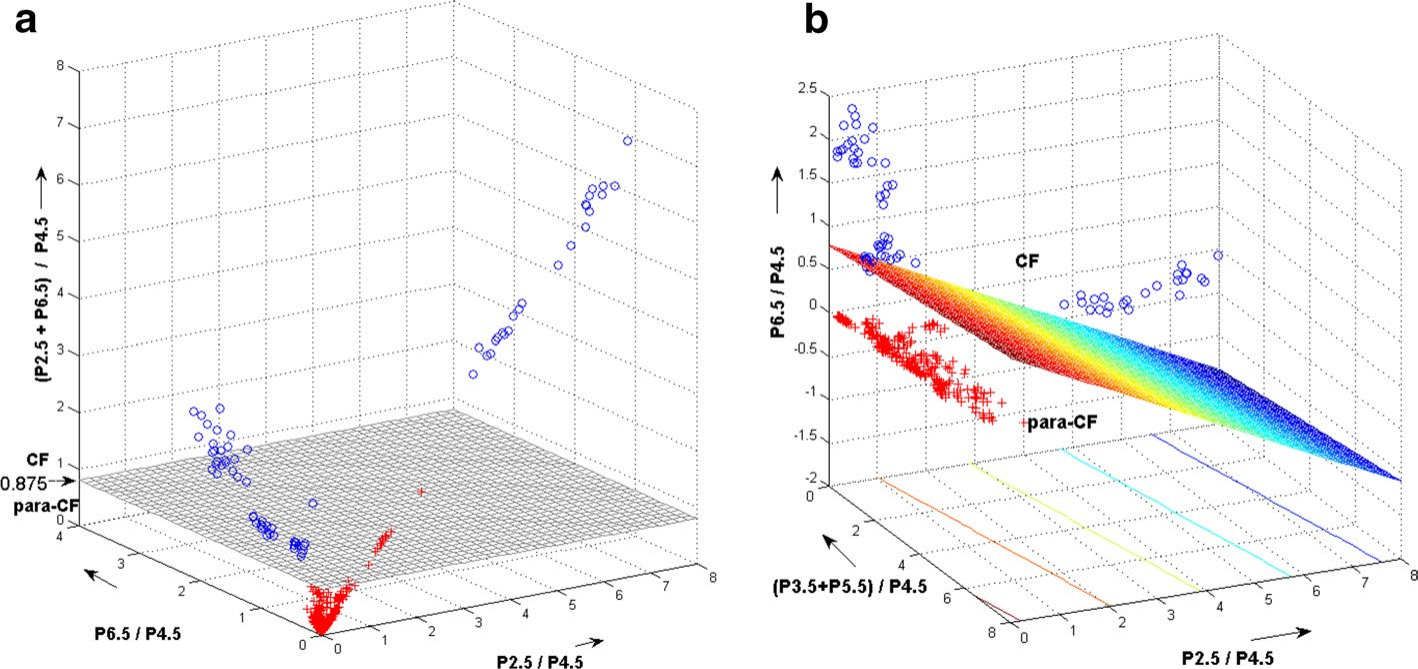
The two methods used to distinguish between central- and paracentral fixation. The *circles* are measurement with central fixation, whereas the crosses stand for paracentral fixation. **a** Simple threshold for (*P*_*2.5*_ + *P*_*6.5*_ *)/P*_*4.5*_ (Method 1). **b** Three-way discriminant analysis (Method 2 for 3D case). Shown is the discriminant plane. **a** shows the threshold for (*P*_*2.5*_ + *P*_*6.5*_)/*P*_*4.5*_, above which the eye is considered to fixate properly. **b** shows the discriminant plane according to Eqs. (4, 5 and 6) that best separates the same measurements in 3D parameter space

Table 1 shows the results for the different methods tested with respect to their ability to differentiate between central fixation and lack thereof. The simple combination of the normalized powers at 2.5 and 6.5 *f*_*s*_ (first column) gave best sensitivity (0.9917) at still a reasonable specificity (0.9625), while the 3- and 4-way discriminant analysis both gave the highest specificity (1.00). Since the PVS was conceived as a screening device, it is expected to miss central fixation as rarely as possible. Therefore, we chose the simple normalized sum of the powers at 2.5 and 6.5 *f*_*s*_ (first column) to be the central fixation criterion, with an adaptively defined threshold of 0.875. Nevertheless, the discriminant functions for the 3D and 4D cases were included as alternative decision making mechanisms in the software, and can be selected from the control panel of the device. Figure 8 shows the plots for the two methods used to distinguish between central- and paracentral fixation. The circles are measurement with central fixation, whereas the crosses stand for paracentral fixation. Figure 8a shows the threshold for (*P*_*2.5*_ + *P*_*6.5*_)/*P*_*4.5*_, above which the eye is considered to fixate properly (simple threshold, Method 1). Figure 8b shows the discriminant plane according to Eqs. (4, 5 and 6) that best separates the same measurements in 3D parameter space.

### Focus curves and FD criterion

The data for the cyclopleged focus curve were obtained between 30 and 60 min after the cyclopentolate 1 % drops were administered to the 10-year-old boy’s eye, and at 60 min the cycloplegic refraction was determined to be +1.00 D with respect to infinity. Thus, *under cycloplegia*, lens power of +1.00 D would have to be added to focus the eye at infinity, and +3.00 D more lens power would have to be added (for a total of +4.00 D) for the eye to be in focus on the target at 33.3 cm.

**Fig. 9:**
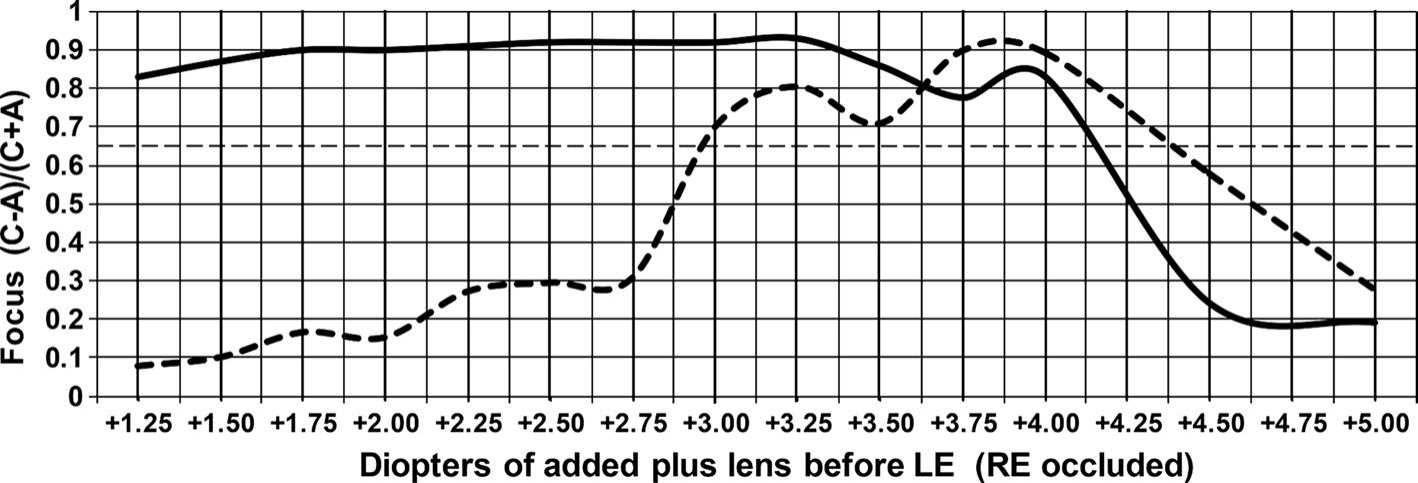
The focus curve of the left eye (LE) of a 10-year old boy, to determine a “passing” threshold for focus. *(C*–*A)/(C* + *A)* is the focus goodness signal. The dashed line was obtained under cycloplegia. Shown is the 0.65 threshold used to discriminate between “passing” and “failing” focus

Figure 9 shows the non-cyclopleged focus curve as a solid line, and the cyclopleged (and dilated) focus curve as a dashed line. Note that the *dashed cyclopleged curve* indeed peaks at +3.87 D, close to +4.00 D. With only 0.50 D of additional plus lens power, for a total of +4.37 D, the normalized focus signal falls to 0.65, the level to which = 1.00 D of extra plus power has previously caused the signal level to fall in older adults, thus illustrating the steepening of the focus curve in the plus direction in the dilated eye.

The peak of the *solid non-cyclopleged curve* is at about +3.25 D of added lens power. Uncorrected hyperopic children such as our 10-year-old boy habitually accommodate to compensate for most of their hyperopic error and cannot easily relax this “tonic” accommodation. If his tonic accommodation is, say, +0.75 D, then instead of +4.00 D of added lens power necessary for his eye to be in focus for the target, only +3.25 D of added lens power is necessary (0.75 + 3.25 = 4.00 D), explaining the peak focus signal in the solid non-cyclopleged curve at +3.25 D of added lens power. In this non-dilated eye, the normalized focus signal level falls to 0.65 at a total of approximately +4.16 D of added lens power, or +0.91 D of additional plus lens power beyond the +3.25 value at best focus. Thus the normalized focus signal level in the non-cyclopleged, non-dilated eye falls to the 0.65 level with almost 1.00 D of blur in the plus direction, helping to establish the 0.65 level as a reasonable threshold level for detecting the desired ± 1.00 D of defocus.

### Device performance on all test subjects

The agreement between the ophthalmologist’s finding (“gold standard”) and the device analysis served as a basis for the evaluation of the instrument. The device gave a binary decision (“pass” or “fail”) for each of the four parameters examined—central fixation (CF) for each eye, and focus detection (FD) for each eye. The instrument was considered to have made a correct decision if the decisions coincided with the ophthalmologist’s findings (“correctly identified”). Table 2 shows the correct (coinciding) and incorrect (non-coinciding) decisions made by the device, separately for the patient and control group and for both, and for the CF and FD function separately and together. Overall, only two of 37 subjects (5.4 %) were misidentified, both of which were false-positive FD. Table 3 shows the same results in terms of true negative (TN), false positive (FP), true positive (TP) and false-negative (FN) readings, along with the sensitivity and specificity. The sensitivity was 100 % for both CF and FD. The specificity for CF was 100 and 89.5 % for FD. The overall sensitivity and specificity were 100 and 94.7 %, respectively.

**Table 2:** Device performance in terms of agreement with the clinical exam

	FD				CF				Both			
	CI	%	ICI	%	CI	%	ICI	%	CI	%	ICI	%
Normals	17	89.5	2	10.5	19	100.0	0	0	17	89.5	2	10.5
Patients	18	100.0	0	0.0	18	100.0	0	0	18	100.0	0	0.0
All	35	94.6	2	5.4	37	100.0	0	0	35	94.6	2	5.4

*CI* correctly identified (coinciding with the ophthalmologist’s finding), *ICI* incorrectly identified (not coinciding with the ophthalmologist’s finding)

**Table 3:** Calculation of the sensitivity and specificity based on the 37 test subjects studied

	TN	FP	TP	FN	Sensitivity (%)	Specificity (%)
	Normals (n = 19)		Patients (n = 18)			
CF	19	0	18	0	100	100.0
FD	17	2	18	0	100	89.5
Both	36	2	36	0	100	94.7

*TN* true negative, *FP* false positive, *TP* true positive, *FN* false negative

## Discussion and conclusion

This is the first clinical validation of our second-generation PVS. The new methods employed, such as spatially modulated polarization using a rotating WP, phase-shift-subtraction, combined central fixation and focus detection using one sensor per eye, synchronization between analog-to-digital conversion, scanning system and power line frequency, greatly increase the signal-to-noise ratio, simplify the hardware, and make the signals largely independent of corneal birefringence. Adding the focus detection feature significantly increases the capability of the device to detect risk for amblyopia.

Central fixation thresholds performed remarkably well on both fixation calibration data and in the subjects studied. On the other hand, confirming focus threshold levels in non-cyclopleged children is complicated by the presence of an unknown amount of tonic accommodation. Nevertheless, the focus signal level of 0.65 appeared to be reasonably correct in the one 10-yr-old child whom we studied extensively. The statistical results presented in Tables 2 and 3 confirm the appropriate choice of the selected thresholds for both central fixation and focus detection. With overall sensitivity of 100 % and specificity of 94.7 %, we believe that the device is suitable for mass screening of children, where high sensitivity is particularly desirable.

## Abbreviations

ADC: analog-to-digital converter
BEPD: “bull’s eye” photodetector
CF: central fixation
CI: confidence interval
CSL: chip support library (Texas instruments)
D: diopter(s)
DIO: digital input–output(s)
DSP: digital signal processor
GUI: graphical user interface
HWP: half wave plate
FD: focus detection
FN: false-negative
FP: false positive
FPGA: field programmable gate array
LED: light emitting diode
MPE: maximum permissible exposure
PVS: pediatric vision screener
PhSS: phase-shift-subtraction
RBS: retinal birefringence scanning, retinal birefringence scanner
TI: Texas instruments
TN: true negative
TP: true positive
WP: wave plate
